# Altered colonic function and microbiota profile in a mouse model of chronic depression

**DOI:** 10.1111/nmo.12153

**Published:** 2013-06-17

**Authors:** A J Park, J Collins, P A Blennerhassett, J E Ghia, E F Verdu, P Bercik, S M Collins

**Affiliations:** * Department of Medicine, Farncombe Family Digestive Health Research Institute, McMaster UniversityHamilton, Canada

**Keywords:** colonic motility, CRH, depression, IBS, microbiome

## Abstract

**Background** Depression often coexists with the irritable bowel syndrome (IBS) which is characterized by alterations in gut function. There is emerging evidence that the microbial composition (microbiota) of the gut is altered in IBS, but the basis for this is poorly understood. The aim of this study was to determine whether the induction of chronic depression results in changes in the colonic function and in its microbial community, and to explore underlying mechanisms.

**Methods** Bilateral olfactory bulbectomy (OBx) was used to induce depression-like behavior in mice. Colonic function was assessed by measuring muscle contractility, pellet excretion, c-fos activity, and serotonin levels. Microbiota profiles were obtained using denaturing gradient gel electrophoresis (DGGE). The hypothalamic-pituitary axis (HPA) was assessed by the hypothalamic expression of corticotropin-releasing hormone (CRH). In separate studies, mice without OBx received CRH via intracerebroventricular (ICV) infusion for 4 weeks prior to assessing colonic function and microbiota profiles.

**Key Results** Olfactory bulbectomy mice demonstrated chronic depression- and anxiety-like behaviors associated with elevated central CRH expression and increases in c-Fos activity, serotonin levels, and motility in the colon. These changes were accompanied by an altered intestinal microbial profile. Central CRH administration produced similar changes in behavior and motility and altered the microbiota profile in the colon.

**Conclusions & Inferences** The induction of chronic depression alters motor activity and the microbial profile in the colon likely via activation of the HPA. These findings provide a basis for linking the behavioral and gastrointestinal manifestations of IBS.

## Introduction

Psychiatric comorbidity, including depression and anxiety, occurs frequently in up to 60% of all patients with a functional gastrointestinal (GI) disorder such as the irritable bowel syndrome (IBS).^1^ Recently, IBS has also been associated with changes in the GI microbiota including reduced diversity^2^–^5^ and temporal instability^6^ at the genus level. Animal studies have shown that perturbation of the intestinal microbiota induces increases visceral pain responses^7^ as well as changes in brain chemistry and behavior.^8^ These observations raise the possibility that the intestinal microbiota may play a role in the expression of IBS by interacting with the gut-brain axis.

Conversely, there is an emerging literature demonstrating the bidirectional nature of microbiota–brain interactions, showing that the brain can influence the microbial composition of the gut. These studies have utilized a variety of stressors including pre- and post-natal stressors,^9^,^10^ as well as deprivation of food, water and bedding,^12^ prolonged restraint stress,^13^ and social disruption.^14^ These changes may be associated with increased susceptibility to opportunistic infections,^9^ or colonization by pathogenic bacteria.^13^ However, the impact of depression on the microbiota has not yet been studied.

There is increasing interest in modeling human depression in the mouse to investigate the underlying neurobiology (for review see Ref. 15) and there is strong evidence implicating stress and activation of the HPA axis in the etio-pathogenesis of depression (for review see Ref. 16). These findings prompted us to exploit a previously described model of depression-like behavior with comorbid anxiety, in which mice exhibit hyperresponsiveness to psychological stressors following the bilateral surgical removal of the olfactory bulb.^17^,^18^ Our results indicate that the induction of anxiety and depression influences the composition of the microbial community in the colon, and suggest that these changes are induced by increased activation of the stress response and disturbance of the microbial habitat via alterations in colonic motility.

## Materials and Methods

### Animals

Studies were performed on female C57BL/6 mice purchased from Taconic (Hudson, NY, USA) at 8–10 weeks of age and housed in a specific pathogen-free unit at the McMaster University Central Animal Facility. Mice were allowed to acclimatize for a minimum of 1 week before any experiments were commenced. All experiments were conducted in accordance with the guidelines of the Canadian Council on Animal Care and received approval from the McMaster University Animal Research Ethics Board. Studies were performed in mice undergoing bilateral olfactory bulbectomy (OBx) or in mice with an indwelling mini pump that administered corticotropin-releasing hormone (CRH) via the intracerebroventricular (ICV) route. A group of OBx mice were subjected to water avoidance stress (WAS). All surgeries were completed at 9–11 weeks of age and all additional measurements were completed at 8–10 weeks postsurgery, at which point there were no significant differences in any of the postsurgical health measures (e.g., weight) between the control and experimental animals. Mice were cohoused with both surgical groups represented in each cage (*n* = 2–3 OBx and 2–3 Sham-operated mice; maximum *n* = 5 per cage). Intracerebroventricular mice were single housed to minimize postsurgical trauma. Experiments were completed with *n* = 5–12 mice per group. None of the mice that survived the surgeries were removed from any of the comparisons.

### Bilateral OBx

Mice were anesthetised with ketamine (150 mg kg^−1^, Bimeda-MTC, Cambridge, ON, Canada) and xylazine (10 mg kg^−1^, Bayer Health Care, Toronto, ON, Canada). The skin covering the frontal bones of the skull was shaved and sterilized, and a small incision was made using a scalpel blade. Two burr holes were made in the skull overlying the olfactory bulbs using a microdrill (steel burr; Meisinger, Centennial, CO, USA). The olfactory bulbs were visualized and removed by aspiration using a blunt glass pipette attached to a vacuum pump. Sham-operated mice received the same treatment except the olfactory bulbs were left intact. The wounds were packed with surgical sponge (gel foam; Pfizer, New York, NY, USA), closed by suture, and treated with antibiotic (Baytril, topical; Bayer Health Care). Following recovery, the mice received subcutaneous saline and an analgesic (buprenorphine 0.05 mg kg^−1^; Reckitt Benckiser, Mississaugua, ON, Canada). Mice were monitored daily for up to 2 weeks postsurgery. All of the OBx surgeries were visually confirmed postmortem, and representative samples were confirmed by histology.

### Intracerebroventricular infusion

This protocol allows for continuous administration of substances into the cerebral spinal fluid bypassing the blood brain barrier. In brief, 28 day micro-osmotic pumps^20^ (Alzet; Durect Corp., Cupertino, CA, USA) were loaded with CRH (50 *μ*g kg^−1^; Sigma-Aldrich, Oakville, ON, Canada) or saline. Mice were anesthetized as above, and an incision was made overlying the skull. A pocket was formed in the skin over the shoulder blades using iris scissors with an angled blade to accommodate the pump. The pumps were attached to a 3-mm-long cannula (Plastics One, Roanoke, VA, USA) using 1.5–2 cm of polyethylene tubing (PE-60; Durect, Cupertino, CA, USA). A microdrill was used to make a small hole in the skull overlying the lateral ventricle, approximately 1.0 mm lateral and 1.0 mm caudal to bregma. This location was identified using a stereotaxic apparatus. After the pump was placed under the skin, the cannula was inserted into the hole and attached to the skull using cyanoacrylate gel (Durect). The incision was closed with suture.

### Behavioral assessment

Olfactory bulbectomy mice were exposed to a battery of tests for anxiety- and depression-like behavior including tail suspension test^21^ (TST), step-down^22^ (SD), and open field^23^ (OF) tests. In the TST, mice were suspended by the tail approximately 50 cm from the base of a retort stand using masking tape, which covered the most distal 1 cm of their tail, a short metal bar, and surgical gut. The test lasted 6 min and duration of immobility was measured as an indicator of depression-like behavior.^21^ In the SD test, mice were individually placed on the center of a wire-mesh covered platform (10 cm diameter, 4 cm high) that rested on a black plexi-glass surface, and the time to step-down on the novel surface with all four paws was recorded for 5 min. Behavior in a novel OF was measured using a fully automated system (arena 27.3 × 27.3 cm; Med Associates Inc., St. Albans, VT, USA). Parameters include: total-distance traveled, percent distance in center zone, and rearing counts. All experiments were conducted in a quiet, well-lit room, with one handler throughout. Pairs of mice, consisting of one OBx and one control sham, were tested at the same time when feasible. Corticotropin-releasing hormone and saline mice were tested using the OF protocol described above. All OF data were generated by Activity Monitor software (product SOF-811; Med Associates Inc.) based on predetermined parameters.

### Stressors

Olfactory bulbectomy has been shown to result in hyperresponsiveness to experimental stressors.^17^,^18^ We exposed a separate group of OBx mice to WAS^24^ to activate the central stress response and examine the enteric response via colonic c-fos and serotonin levels. Briefly, OBx mice were taken directly from their home cage and placed on 2 cm high platforms surrounded by water (1 cm deep) for 10 min immediately before they were euthanized by cervical dislocation.

### Isolation of the paraventricular nucleus of the hypothalamus

Whole brains were carefully removed upon euthanization and immediately frozen in isopentane (Sigma-Aldrich) cooled to −80 °C using dry ice. Brains were stored at −40 °C until they were transferred to a microtome cryostat with a chamber temperature of −20 °C (Microm 550; Thermoscientific, Nepean, ON, Canada). Brains were placed in Tissue-Tek O.C.T. Compound (Sakura Finetek, Torrance, CA, USA) and positioned for coronal sections. The brains were trimmed at a thickness of 80 *μ*mol L^−1^ until the suprachiasmatic nucleus. Next, slices were cut at 8 *μ*m and every tenth slice was placed on a slide and stained with toluidine blue to visually identify the start of the paraventricular nucleus (PVN). Once this point had been identified, one 100 *μ*m section was removed and a square area containing the PVN was manually excised using a razor blade, and placed in a sterile microtube cooled to −20 °C. An 8 *μ*m section was cut and stained to confirm location, and then a second 100 *μ*m section was taken and cut as before. A final 8 *μ*m slice was stained to ensure that the entire section of tissue collected was in fact PVN. Tissue was frozen at −40 °C until processed.

### RNA isolation and real-time PCR

RNA was isolated from the brain tissue using RNeasy Mini Kit (Qiagen, Mississauga, ON, Canada) and treated with RNase-free DNase (Qiagen) to remove the contaminating genomic DNA. Reverse transcription was performed using 1 *μ*g of RNA and M-MLV Reverse-Transcriptase (Invitrogen, Burlington, ON, Canada). PCR efficiency and optimal annealing temperature for each of the primer pairs were tested via standard curve and thermal gradient experiments, respectively, using a MyiQ2 Real-Time PCR Detection System (BioRad, Mississauga, ON, Canada). The PCR condition used were as follows: 95 °C for 3 min, followed by 40 cycles of 95 °C for 10 s, 58 °C for 30 s, and 72 °C for 30 s. Amplification was performed in triplicate 20 *μ*L reactions using iQ SYBR Green Supermix (BioRad), and normalized to glyceraldehyde-3-phosphate dehydrogenase (GAPDH) expression. The product size was confirmed by gel electrophoresis and the specificity of the amplification was tested by performing melt-curve analysis for all reactions. Corticotropin-releasing hormone primer sequence: forward 5′-CAA CAG GAA ACT GAT GGA GAT T-3′; reverse 5′-GGA GCT GCG ATA TGG TAC AG-3′. Glyceraldehyde-3-phosphate dehydrogenase primer sequence: forward 5′-CCA TGG AGA AGG CTG GGG; reverse 5′-CAA AGT TGT CAT GGA TGA CC. Gene expression was analyzed using the 2^−ΔΔCt^ method.

### Bacterial profiling

Stool samples were collected by restraining the mice and collecting the first fresh fecal pellet passed into a sterile microtube, which was immediately frozen in liquid nitrogen. Bacterial DNA was extracted using a Phenol/chloroform/isoamyl method, paired with a Clean and Concentrator kit (Zymo Research Corp., Irvine, CA, USA). PCR was performed using HDA-1 and HDA-2 universal primers for the V3 region of the 16s rRNA gene. The PCR products (rDNA) were separated based on their G+C content and distribution using denaturing gradient gel electrophoresis (DGGE). Gels (35–55% denaturing gradient) were subjected to a constant voltage of 130 V for 4.5 h at 60 °C using a Dcode universal mutation detection system (Bio-Rad Laboratories, Richmond, CA, USA.). After electrophoresis, the gels were stained for 20 min in 1× TAE containing SYBR Green (Molecular Probes, Eugene, OR, USA), photographed under UV illumination, and analyzed using Bionumerics software (Applied Maths, Austin, TX, USA). The Pearson correlation coefficient was used as it accounts for both presence/absence of a band as well as the band intensity.^25^,^26^ Statistical analysis was completed by running a script in the Bionumerics software that compares the within- and between-group similarities with randomized tests (1000 iterations).^28^ The *P* value represents how frequently the random similarity is greater than the experimental similarity; *P* < 0.05 represents a significantly distinct group. The dendrograms were constructed using the neighbor-joining clustering method. This method builds a tree based on the principle of minimum evolution by starting with the two most similar samples, and then creates a branching pattern by adding branches of the shortest possible length (smallest amount of change between samples) until all individuals are included. It does not assume a constant rate of evolution/change as Unweighted Pair Group Method with Arithmetic Mean (UPGMA) does, and is considered to be more realistic and consistent.^28^

### Fecal output

Fecal output (FO) has been used as an indicator of colonic motility^29^ and was measured in a group of OBx mice subjected to WAS. Five separate WAS experiments were conducted simultaneously in an attempt to equalize handling-stress on grouped housed OBx and sham-operated mice. Output was assessed by counting the total number of fecal pellets expelled during a 10 min session in an OF arena.

### Colonic muscle contractility

Olfactory bulbectomy and control mice were taken directly from their home cage and euthanized by cervical dislocation. Sections of distal colon were removed and placed in Krebs buffer containing (in mmol L^−1^) 120.9 NaCl, 1.2 NaH_2_PO_4_, 15.5 NaHCO_3_, 5.9 KCl, 2.5 CaC1_2_, 1.2 MgC1_2_, and 11.1 glucose. As previously described,^30^ two pieces of 5–10 mm silastic tubing (0.94 mm; Dow Corning, Midland, MI, USA) were placed in the lumen at either end of the tissues to allow for proper oxygenation. A closed end loop of suture was placed at one end and attached to a tissue hook in the organ bath containing Krebs buffer at 37 °C under 95% O_2_–5% CO_2_. The other end of the tissues were tied with a long suture and attached to a force displacement transducer, which was then calibrated to a tension of 1 g. The tissues were then stretched to optimal tension (800 mg) and then allowed to equilibrate for 30 min. Tension change in response 10^−7^ log M (mol L^−1^) of a cholinergic agonist (carbamoylcholine chloride; Sigma-Aldrich) was recorded using Power Laboratory (AD Instruments, Hastings, UK) and normalized to cross sectional area (CSA). Cross sectional area was calculated as follows: CSA (mm^2^) = tissue wet weight (mg)/[(length of tissue (mm) × density (mg mm^−3^)] where the density of smooth muscle was assumed to be 1.05 mg mm^−3^.

### Cytokine profiles

Sections of colon and mesenteric lymph nodes (MLN) from OBx mice were removed upon euthanization, placed in a sterile microtube, and immediately frozen in liquid nitrogen. Tissue was stored at −80 °C until homogenization in Tris HCl buffer containing protease inhibitors (Sigma-Aldrich). Cytokine levels in the supernatant were measured using a BD Cytometric Bead Array (mouse inflammation kit; BD Biosciences, Mississauga, ON, Canada) as per the manufactures instructions. Results were analyzed using BD FACSArray Bioanalyzer System (BD Bioscience).

### c-Fos activation in colonic nerves

Olfactory bulbectomy mice were exposed to 10 min of WAS prior to euthanization. Tissues were frozen in Tissue-Tek O.C.T. Compound (Sakura Finetek, Tokyo, Japan) and stored at −40 °C. Distal colons were sectioned to 10 *μ*m and mounted onto double frost slides (Surgipath, Winnipeg, MB, Canada) and stored at −20 °C until processing. Briefly, slides were thawed at room temperature and rinsed in PBS (pH 7.4) to remove residual embedding compound and then fixed by incubating in a 10% buffered formalin solution (VWR, Radnor, PA, USA) for 20 min, followed by several washes in PBS. Endogenous proteins were blocked by incubating slides in a 4% normal goat serum block with 0.4% Triton-X (Sigma-Aldrich). Antigen was then detected using an overnight incubation with monoclonal rabbit anti-c-fos antibody (1 *μ*g mL^−1^; Cell Signaling, Danvers, MA, USA). Primary antibody was detected using an Alexa Fluor 488 goat antirabbit secondary antibody (1 : 200, Abcam, Cambridge, MA, USA) for 2 h. Slides were then washed in PBS and coverslipped with aqueous mounting medium (Vector Labs, Burlington, ON, Canada). Images from stained sections were captured using an Olympus BX51 fluorescence microscope (Olympus, Mississauga, ON, Canada), and QImaging micropublisher 3.3 RTV camera (QImaging, Surey, BC, Canada). Images were analyzed using Image Pro 6.3 (Media Cybernetics, Bethesda, MD, USA) and ImageJ computer software (National Institutes of Health, Bethesda, MD, USA). Briefly, threshold limits were set to detect and quantify only c-fos positive fluorescent staining. Positive staining as a percentage of total tissue area was measured using Image-J measurement analysis.

### Colonic serotonin

Olfactory bulbectomy mice were exposed to 10 min of WAS prior to euthanization. Sections of colon were removed, placed in a sterile microtube, and immediately frozen in liquid nitrogen. Tissues were homogenized in acetic acid and centrifuged. Serotonin levels in the supernatant were assessed using a commercially available ELISA kit (Rocky Mountain Diagnostics, Colorado Springs, CO, USA).

### Data analysis

Specific analysis of DGGE results was described above. All other experiments were tested using GraphPad Prism (GraphPad software, La Jolla, CA, USA). Comparisons were made using unpaired Student's *t*-tests. A modified *t*-test was used for data sets that displayed significantly different variances (Welch correction), and non-parametric test was used for data with non-Gaussian distributions. The significance level was set at *α* = 0.05.

## Results

### Behavioral responses in OBx mice

**Figure 1:**
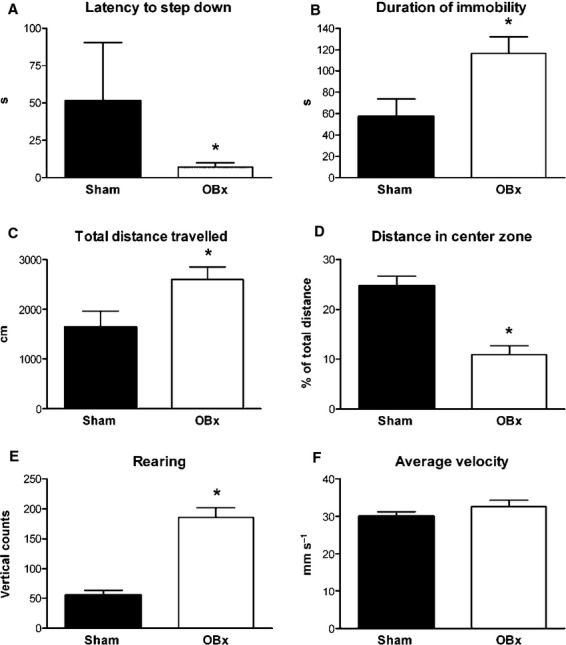
Behavioral profile of olfactory bulbectomy (OBx) Mice. (A) Olfactory bulbectomy mice step-down quicker than sham-operated controls. (B) Olfactory bulbectomy mice have a higher duration of immobility in the tail-suspension test indicative of higher levels of behavioral depression. Olfactory bulbectomy mice show elevated levels of anxiety-like behavior in an open field test as shown by (C) hyperlocomotion, (D) decreased distance travelled in the anxiety-inducing center zone, and (E) increased rearing. (F) Olfactory bulbectomy does not affect general measures of motor activity. (*P <* *0.05 *vs* sham controls). Data are presented as mean ± SEM.

Olfactory bulbectomy mice demonstrated a significant reduction in the latency to step down test compared with sham-operated controls (Fig. 1A) and showed prolonged immobility in the tail suspension test (Fig. 1B). Olfactory bulbectomy mice also demonstrated hyperlocomotion (Fig. 1C) and anxiety-like behaviors, as reflected by reduced exploratory activity (Fig. 1D) and increased rearing (Fig. 1E) in the OF test. As shown in Fig. 1F, there was no alteration in the average velocity of locomotion. These results are consistent with a profile of depression- and anxiety-like behaviors and are in agreement with the previously described behavioral profile of this model.^17^,^18^

### Corticotropin-releasing hormone expression in OBx mice

**Figure 2:**
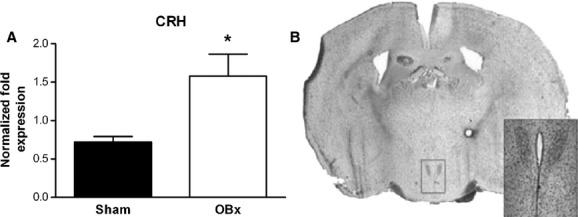
Hypothalamic corticotropin-releasing hormone (CRH) expression in Olfactory bulbectomy (OBx) mice. (A) Olfactory bulbectomy mice show increased expression of CRH mRNA in the paraventricular nucleus of the hypothalamus. (*P <* *0.05 *vs* sham controls). Data are presented as mean ± SEM. (B) Photo shows the location of the paraventricular nucleus of the hypothalamus. Inset box indicates the area that was isolated for analysis of CRH expression.

As shown in Fig. 2, OBx mice displayed a significant twofold increase in the basal expression of CRH in the PVN of the hypothalamus, suggesting heightened activation of the HPA axis following surgery.

### Microbiota profile in OBx mice

**Figure 3:**
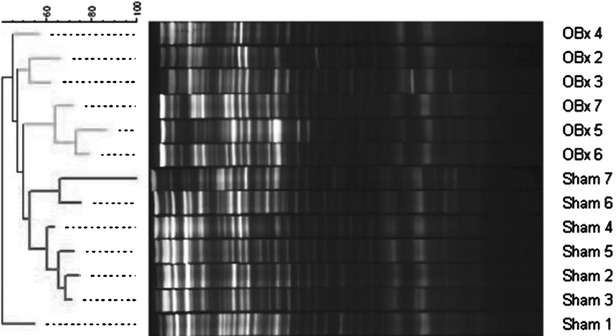
Microbiota profile in Olfactory bulbectomy (OBx) mice. Cluster analysis of the fecal microbiota at 8 weeks postsurgery shows a clear separation between OBx and sham mice. The similarity matrix for this data indicated a 60.4% and 60.9% similarity within the sham and OBx groups, respectively, and 49.1% similarity between the two groups. Branch distances indicate% similarity between individuals.

As shown in Fig. 3, cluster analysis of the banding patterns was used to compare microbial profiles in OBx and sham-operated mice. The similarity matrix created using Pearson's correlation coefficient (PCC) indicated a 60.4% and 60.9% similarity within the sham and OBx groups, respectively, but only a 49.1% similarity between the two groups. Permutation testing indicated that the profiles seen in OBx and sham-operated mice were significantly different (*P* < 0.05). The use of PCC, which accounts for band intensity as well as presence/absence,^26^–^27^ suggested that the difference was mainly due to a change in the proportion of certain bacterial phyla rather than the appearance or disappearance of bacteria at the phylum level following OBx. These findings indicate that OBx results in a redistribution of the relative abundances of bacterial phyla within the intestinal microbiota.

### Inflammatory cytokine expression in OBx mice

We observed no differences in the levels the pro-inflammatory cytokines IL-1β, TNF-α, IL-6, and IL-17, the chemokine MCP-1, or anti-inflammatory IL-10 in whole colon tissue or MLN (Table 1). This suggests that OBx did not have a significant effect on baseline production of inflammatory markers in the tissue in close proximity to the microbiota.

**Table 1:** Cytokine levels in colonic wall and MLN of OBx and sham-operated mice (pg mg^−1^ protein)

	Sham MLN	OBx MLN	Sham colon	OBx colon
IL-1β	12.20 ± 1.67	10.97 ± 0.63	2.39 ± 0.30	2.33 ± 0.53
TNF-α	4.90 ± 0.50	5.95 ± 0.40	1.74 ± 0.12	1.74 ± 0.38
IL-6	18.21 ± 1.84	16.58 ± 1.75	5.72 ± 0.46	5.49 ± 0.79
IL-17	12.13 ± 1.38	13.65 ± 1.54	3.24 ± 0.23	2.45 ± 0.40
MCP-1	ND	ND	0.13 ± 0.01	0.15 ± 0.04
IL-10	0.43 ± 0.15	1.05 ± 0.32	0.35 ± 0.10	0.48 ± 0.12

OBx, olfactory bulbectomy; MLN, mesenteric lymph nodes.

### Colonic motility in OBx mice

**Figure 4:**
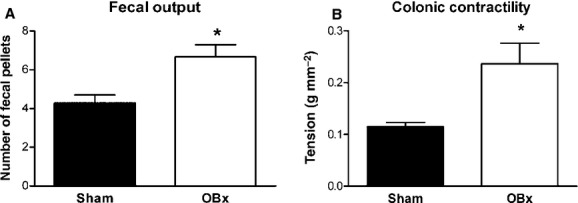
Fecal output (FO) and colonic muscle contractility in olfactory bulbectomy (OBx) mice. (A) Olfactory bulbectomy mice show increased FO when exposed to a mild stressor. (B) Longitudinal smooth muscle from OBx mice generates significantly higher contractile forces in response to a cholinergic agonist. (*P <* *0.05 *vs* sham controls). Data are presented as mean ± SEM.

We next examined colonic motility using two approaches. Firstly, we measured FO as an indicator of stress-induced colonic transit. Exposure to a mild stressor resulted in a significant increase in the number of fecal pellets expelled during the 10 min testing period (Fig. 4A) in OBx mice compared with controls. We used an *in vitro* organ bath method to assess the contractile force of colonic longitudinal smooth muscle in OBx and sham mice. The colon from OBx mice generated a significantly greater force of contraction, compared with controls (Fig. 4B). These results indicate that colonic motility is altered in OBx mice, thus perturbing the habitat of the microbiota.

### Colonic c-Fos expression and 5-HT levels in OBx mice

**Figure 5:**
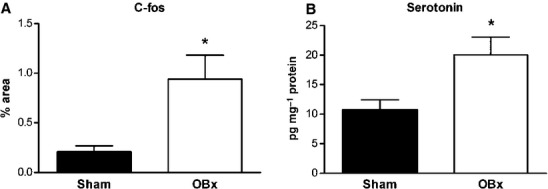
Colonic c-fos expression and 5-HT levels in the colon of Olfactory bulbectomy (OBx) mice. Olfactory bulbectomy mice have higher levels of (A) c-fos protein and (B) serotonin in the colon after exposure to a mild stressor (10 min water avoidance stress; *P < 0.05). Data are presented as mean ± SEM.

As shown in Fig. 5A, immunohistochemistry revealed an almost fourfold increase in the c-fos protein in the myenteric plexus of OBx mice after exposure to a mild stressor. We next investigated changes in colonic 5-HT, an important regulator of GI motility. As shown in Fig. 5B, levels of serotonin were significantly higher in colonic tissue from OBx mice compared with sham-operated controls.

### Effect of ICV administration of CRH in mice (without OBx)

**Figure 6:**
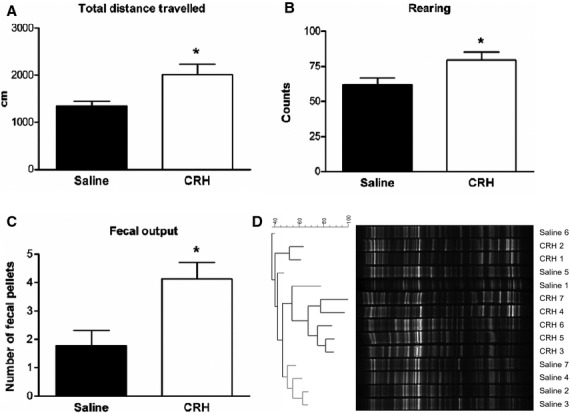
Central administration of corticotropin-releasing hormone (CRH) affects behavior and gastrointestinal function. Mice treated with CRH have higher levels of anxiety-like behavior as shown by increased (A) locomotion and (B) rearing compared with saline-treated controls. (C) Corticotropin-releasing hormone mice show increased fecal pellet output in an open field (*P < 0.05). Data are presented as mean ± SEM. (D) Twenty-eight days of CRH administration (ICV infusion) resulted in a significantly different bacterial profiles in CRH-treated mice compared with saline-treated controls (*P* < 0.05). Denaturing gradient gel electrophoresis profiles showed 79.4% and 65.7% similarity within the saline and CRH groups, respectively, and 68.0% similarity between the two groups.

The next series of experiments were conducted in mice without OBx but that were receiving i.c.v CRH over 28 days. Micro-infusion of CRH into the central nervous system of intact mice resulted in significant changes in OF behavior. Greater locomotion and more rearing, as was observed in OBx mice, were also evident in CRH-treated mice compared with controls (Fig. 6A and B). Corticotropin-releasing hormone-treated mice also exhibited greater fecal pellet expulsion during the 10 min OF session compared with controls (Fig. 6C). Twenty-eight days of CRH administration also altered the colonic bacterial profile compared with saline-infused mice. The similarity matrix indicated a 79.4% and 65.7% similarity within the saline and CRH groups, respectively, and 68.0% similarity between the two groups. Permutation test revealed that the profile from the CRH mice differed significantly from that of the saline control group (*P* < 0.05).

## Discussion

Olfactory bulbectomy is a widely used animal model of depression with comorbid anxiety and in which there is hyperresponsiveness to experimental stressors.^17^,^18^ Both OBx rats and mice display behavioral abnormalities that are independent of postsurgical anosmia and are reversed by several classes of antidepressants.^18^ The behavioral profile of OBx mice seen in this study includes increased activity and rearing in a novel environment and these behaviors have been attributed to a loss of habituation. Prolonged immobility seen in the tail suspension test is a sign of helplessness and indicative of depression.^21^ Taken together, these findings are consistent with those in the literature and support the use of the OBx mouse as a model of depression- and anxiety-like behavior.^17^,^18^

The role of immune activation and inflammation in the pathogenesis of depression, although controversial, is gaining ground based on both animal and human data.^31^ The linkage is based in part on small elevations in cytokines and other inflammatory markers in a subgroup of patients with depression, as well as on the behavioral consequences of cytokine administration (for reviews see Ref. 32–33). In our study, we did not find an increase in the range of cytokines. Given that evidence of inflammation or immune activation occurs in only a subgroup of patients with depression and is not therefore critical for the expression of depression-like behavior, we assume the behavioral profile of OBx mice is independent of peripheral inflammatory or immunological processes. However, we acknowledge that the cytokine profile seen in our study may also reflect impaired cytokine secretion in response to lipopolysaccharide that was reversed following prolonged treatment with tricyclic antidepressants in OBx rats.^34^

It should be acknowledged that alterations in food consumption could theoretically influence the composition of the gut microbiota in some models of psychological perturbation. We contend that altered feeding behavior does not play a role in the changes in microbiota seen in this model. Earlier studies by Meguid *et al*. indicated that while there were qualitative changes in feeding patterns at day 14 post surgery, the quantity of food consumed was not affected following surgery.^35^ Furthermore, additional studies did not show any chronic (40 days) changes in either qualitative or quantitative feeding behavior.^36^ Consistent with these studies, we found no changes in bodyweight in OBx mice.

Depression in humans is often accompanied by changes in colonic transit^37^ and our study clearly demonstrates the OBx mice have altered colonic motility that is evident both *in vivo* and *in vitro*. The observation of altered colonic transit and a shift in the profile of the microbiota is consistent with the findings of Rodes *et al*. who showed changes in the colonic transit altered the composition and stability of the microbial community in the colon.^38^ It is also consistent with changes in colonic physiology and morphology demonstrated in two non-surgically induced models of depression and stress-induced anxiety.^39^ Our study also demonstrates increases in the prokinetic neuropeptide and gut hormone, serotonin in the colonic wall as a putative mediator of the altered motility.

An important finding in this study is the demonstration that experimentally induced depression is accompanied by a shift in the relative abundance of the resident microbes in the gut. This builds on findings in the model of maternal deprivation in which the deprived offspring develop anxiety- and depression-like behavior as well as stress-induced behaviors (for recent review see Ref. 40). Maternal deprivation in mice results in increased susceptibility to inflammatory stimuli in the gut and this could be prevented by prior treatment with antidepressants.^41^ The basis for this increased susceptibility may be due to alterations in the intestinal microbiota as previously shown by Bailey *et al*. in maternally deprived rhesus monkeys.^9^–^10^ However, the basis for the altered bacterial composition of the gut in the model of maternal separation is uncertain and may reflect an abnormal pattern of microbial colonization of the gut due to interruptions in maternal-infant contact in early life. In contrast, interference with colonization during that critical postnatal period is not a concern in the OBx model of depression-like behavior. Our findings indicate that depression leads to a change in the relative abundances of the commensal bacteria in the gut, and is most likely due to a change in colonic physiology altering the habitat for commensal bacteria.^38^

It is possible that the altered intestinal microbiota profile in OBx mice contributes to its behavioral phenotype. A previous study from this laboratory showed that shifts in the relative abundance of intestinal bacteria resulted in changes in the expression of brain-derived neurotropic factor in the hippocampus and changes in behavior.^8^ As we propose that the alterations in the microbiota in OBx mice are secondary to changes in colonic physiology, they are unlikely to represent a microbiota profile that is specific for depression-like behavior.

Activation of the HPA is a feature of the neurobiology of depression; elevated concentrations of CRH have been found in the cerebrospinal fluid of depressed patients^42^ and the PVN of the hypothalamus has been shown to contain increased numbers of CRH containing neurons.^43^ In our study, we found increased expression of CRH in the PVN region of the hypothalamus in OBx mice. In addition, administration of exogenous CRH in normal mice reproduced in them both the behavioral changes and stress-induced changes in colonic motility seen in OBx mice, suggesting a critical role for this peptide in our model. This is supported by the demonstration that central administration of the CRH-2 receptor agonist stimulated colonic transit, but had no effect when administered intravenously to rats.^44^ Taken in conjunction with the present study, these findings link central mechanisms relevant to stress and depression with changes in the intestinal microbiota that are likely secondary to changes in colonic physiology, and support the notion of a bidirectional microbiota–gut–brain axis.^45^–^46^

The results of this study are relevant to our understanding of the frequent coexistence of depression and functional GI disorders such as IBS.^1^ Our findings suggest that comorbid depression is likely to compound the severity of gut dysfunction via depression-induced changes in colonic motility and to further destabilize the intestinal microbiota, resulting in the previously documented temporal instability of the microbiota in patients with IBS.^6^ The destabilization of the microbiota may, in turn, contribute not only to gut dysfunction,^7^ but also to behavioral changes^8^ and hence to the psychiatric comorbidity that is common in IBS patients.^1^ These observations support the clinical investigation of the microbiome in IBS patients that demonstrated statistically significant associations between the microbial composition of the gut with clinical depression and with slow colonic transit.^47^

## Funding

This work was supported by grants in aid from the Canadian Institutes of Health Research (CIHR), and the Crohn's and Colitis Foundation of Canada (CCFC) to S. M. Collins and P. Bercik. J. E. Ghia was the recipient of a fellowship award from CIHR and the Canadian Association of Gastroenterology. E. F. Verdu holds a Canada Research Chair. P. Bercik is a recipient of HHS Research Early Career Award.

## Disclosure

The authors have no competing interests.

## Author Contribution

AJP & SMC design of research study and writing of paper; AJP, JC analysis of data; EFV, JC, JEG, PB contributed essential reagents or tools.

## Abbreviations

CRH: corticotropin-releasing hormone
DGGE: denaturing gradient gel electrophoresis
GI: gastrointestinal
IBS: irritable bowel syndrome
OBx: olfactory bulbectomy
OF: open field
PCC: Pearson's correlation coefficient
PCR: polymerase chain reaction
SD: step down
TST: tail-suspension test
